# Targeted RNA sequencing reveals differential patterns of transcript expression in geographically discrete, insecticide resistant populations of *Leptinotarsa decemlineata*

**DOI:** 10.1002/ps.6393

**Published:** 2021-05-03

**Authors:** Justin Clements, Kurt Lamour, Kenneth Frost, James Dwyer, Anders Huseth, Russell L Groves

**Affiliations:** ^1^ Department of Entomology, Plant Pathology, and Nematology University of Idaho Parma ID USA; ^2^ Department of Genome Science and Technology University of Tennessee Knoxville TN USA; ^3^ Department of Botany and Plant Pathology Oregon States University Corvallis OR USA; ^4^ Cooperative Extension University of Maine Orono ME USA; ^5^ Department of Entomology and Plant Pathology North Carolina State University Raleigh NC USA; ^6^ Department of Entomology University of Wisconsin‐Madison Madison WI USA

**Keywords:** Colorado potato beetle, targeted RNA sequencing, insecticide resistance

## Abstract

**BACKGROUND:**

The Colorado potato beetle (*Leptinotarsa decemlineata* Say) is a major agricultural pest of commercial potatoes, partially due to its ability to rapidly develop resistance to multiple insecticide modes of action. Patterns of *L. decemlineata* insecticide resistance in the contiguous United States have been linked to geographic location and regional management practices. Several previous studies have classified enzymes that are overexpressed following *L. decemlineata* exposure to commercial pesticides, many of which have been linked to xenobiotic metabolism. Studies have further associated geographic disparities in resistance patterns to cross‐resistance driven by fungicide exposure in the East Coast and Midwest.

**RESULTS:**

In this study, our objective was to investigate transcript expression of 38 previously classified detoxification enzymes induced by imidacloprid (an insecticide) and chlorothalonil (a fungicide) within five discrete populations of *L. decemlineata* obtained from areas in the USA representing eastern, midwestern and western production regions. We found unique patterns of transcript expression in different geographic locations, including overexpression of transcripts related to insecticide metabolism within insecticide‐resistant populations.

**CONCLUSION:**

The results suggest the genetic response of these populations may be partially linked to geographic location and corresponding management practices. © 2021 The Authors. *Pest Management Science* published by John Wiley & Sons Ltd on behalf of Society of Chemical Industry.

## Graphical abstract

Median lethal dose assays and targeted RNA sequencing demonstrated that geographic location and management practices may influence transcript expression of insecticide resistance mechanisms in distinct populations Colorado potato beetles.

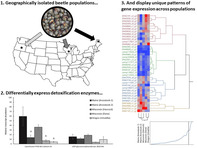

## 1 INTRODUCTION

Management of agricultural crops relies on integrated pest management strategies to mitigate production losses due to insect infestation.^1^ Producers of field and vegetable crops depend on local and regional guidelines for pesticide applications to limit insect infestation and minimize associated damage.^1^ Within an agroecosystem, a producer may need to manage multiple insect, pathogen and plant species resulting in the application of several insecticide, fungicide and herbicide mode of action groups, respectively.^2^ Pesticide applications used for the same cropping system can vary among producers and between different geographic regions. One pest of solanaceous crops that is often controlled using a diverse set of cultural and chemical management practices is the Colorado potato beetle (*Leptinotarsa decemlineata* Say).^3^ If not properly managed, this specialist herbivore can cause significant defoliation of the potato canopy that can result in yield loss and economic injury for the producer.^4^, ^5^ In addition to its ability to rapidly defoliate plants, *L. decemlineata* is considered a major pest species because of its ability to develop resistance to insecticides at a rapid rate.^6^ To combat resistant populations, *L. decemlineata* can be effectively managed using combinations of cultural management strategies including crop rotation in space and time, as well as trap crops, but insecticidal strategies remain the most common control measure for this pest.^1^, ^3^

Insecticide resistance develops and propagates within insect populations through the selection of individuals which possess genetic attributes that, when passed onto offspring, result in resistant phenotypes.^7^ Insecticide resistance can develop through multiple mechanisms, including enhanced metabolic breakdown of insecticides, reduced cuticular penetration, target site insensitivity, and behavioral resistance or avoidance.^7^, ^8^ Previous *L. decemlineata* investigations have classified possible mechanisms of insecticide resistance within select populations, including the detoxification and removal of insecticides from the insect's body through phase 1 (detoxification through breakdown) and phase 2 (removal through excretion) enzymes.^9^, ^10^, ^11^, ^12^, ^13^, ^14^, ^15^ These studies have examined the overexpression of transcripts corresponding to xenobiotic resistance mechanisms within select populations of *L. decemlineata* classified as insecticide resistant through traditional dose–response bioassays. Crossley *et al*. examined single nucleotide polymorphisms (SNPs) from thoracic muscle tissue and classified SNP‐based markers associated with insecticide resistance.^11^ These studies have identified a multitude of candidate genes that correlate with insecticide resistance. The findings of these studies suggest that a combination of detoxification and excretion enzymes play a significant role in insecticide resistance.^9^, ^10^, ^11^, ^12^, ^13^, ^14^, ^15^ It is also important to note that behavioral resistance and mutations in the nicotinic acetylcholine receptor (the target of the class 4A insecticides) have also been correlated with insecticide resistance.^16^, ^17^, ^18^

Defining the mechanisms of insecticide resistance development within *L. decemlineata* is difficult because of the differences observed between geographically distinct populations of the insect. *Leptinotarsa decemlineata* populations are found within all major potato production areas of the USA. However, insecticide resistance to common insecticide mode of action groups has predominantly been associated with potato production areas on the East Coast and in the Midwest, but not in the Pacific Northwest region.^19^, ^20^, ^21^ Although estimates of total insecticide applications (kg active ingredient [ai] ha^−1^) are similar across the USA, the amount of other pesticides applied differs dramatically in different geographic regions. This is particularly true for the Pacific Northwest, where fields have been historically treated with one‐third (kg ai ha^−1^) of the fungicide input compared with fields in the Midwest and East Coast.^20^ A transcriptomic experiment in which insects from a laboratory population were exposed to both imidacloprid (insecticide; mode of action group 4A, IRAC‐online, v. 9.3, https://www.irac-online.org/, sourced 15 January 2020), and chlorothalonil (fungicide; mode of action code M05, https://www.frac.info/docs/, sourced 15 January 2020), and the transcriptomic response and classification of the enzymatic detoxification mechanisms revealed that the insecticide and fungicide induced similar detoxification mechanisms within individuals from the same population.^10^ This result suggests that the geographic disparity in observed resistance levels can be partially explained by the development of cross‐functional detoxification pathways driven by chronic exposure to both insecticides and fungicides.^10^

In this study, our goal was to investigate the similarities and differences in constitutively overexpressed transcripts among geographically distinct populations of *L. decemlineata*. Geographic location is often related to patterns of pesticide management strategies and chemical inputs which are required for crop protection, and consequently may play a significant role in insecticide resistance development. Examining the patterns of transcript expression of previously classified detoxification mechanisms through targeted RNA sequencing provided insight into transcript regulation corresponding to putative insecticide resistance that were linked to geographic location. Additionally, differences between the overexpression of detoxification mechanisms within resistant populations of *L. decemlineata* may suggest that insecticide resistance can develop in different ways. We hypothesized that the activation of enzymatic detoxification mechanisms may be associated with geographic locations and predicted that overexpression of the classified detoxification mechanisms would be related to conventionally managed fields in the Midwest and East Coast where intensive insecticide and fungicide use co‐occur, when compared with the Pacific Northwest.

## 2 MATERIALS AND METHODS

### 2.1 Insect collections

Newly emerged, overwintered adult *L. decemlineata* and their respective egg masses were collected from potato fields in three major potato regions in the USA: Eastern, Midwestern and Pacific Northwest. A description of the respective insecticide management practices (organic or conventional standard pesticides) for each field location were also acquired from the agricultural producers. Two populations of insects were collected from an agricultural producer located in Aroostook County, Maine representing the Eastern region. The first population, designated as Maine (Aroostook‐1), was collected from organically managed potato with crop inputs consistent with National Organic Program (NOP) standards (https://www.ams.usda.gov/rules-regulations/organic, sourced 15 January 2020). The second population, designated as Maine (Aroostook‐2), was collected from conventionally managed, commercial potato. Two populations of *L. decemlineata* were collected from Wisconsin. The first population was collected from Hancock County, Wisconsin and designated as Wisconsin (Hancock). The producer for this field location used conventional management inputs for potato production. A second population was collected from Dane County, Wisconsin, and designated as Wisconsin (Dane), and this producer used NOP standards. Finally, a single population of *L. decemlineata* was collected from Umatilla County, Oregon and designated as Oregon (Umatilla). The producer supplying this population used conventional management inputs for potato production. Adult insects (*n* = 50) from each field location were hand‐collected, placed in sterile plastic containers with foliage, and shipped overnight to Madison, Wisconsin. Upon arrival, insects were placed into a labeled mesh cage and allowed to freely feed on pesticide‐free potato plants. Mesh cages were placed in an incubator at 26°C, 70% relative humidity (RH), and a 16:8 h light/dark photoperiod. Egg masses were also acquired from producers (*n* = 30). Upon arrival, egg masses were placed in sterile Petri dishes and monitored daily for egg hatch. After egg hatch, larvae were fed untreated foliage and the instar was noted daily. Petri dishes were placed in an incubator held at 26°C, 70% RH, and a 16:8 h light/dark photoperiod.

### 2.2 Median lethal dose assays

To establish median lethal dose (LD_50_) estimates for each experimental population, egg masses obtained from each location were hatched and raised to the second instar stage of development. Larval age was determined according to Boiteau *et al*.^22^ and was used to obtain LD_50_ estimates from feeding bioassays using a range of serial doses of technical imidacloprid solubilized in acetone. A single 0.13‐cm^2^ leaf disk (cultivar Russet Burbank) was placed on a damp sponge covered by a small, circular piece of Whatman filter paper in 12‐well Falcon microplates. The sponge and filter paper were arranged to take up approximately half of the volume of each well and were used to maintain a high level of humidity. A single second‐instar *L. decemlineata* was placed into each well. Immediately prior to placement of the insect, replicate sets of 1 μl aliquots of imidacloprid in acetone ranging between 0 to 170 ng of imidacloprid (Bayer Crop Science) were applied to leaf disks. The same concentrations were used for the bioassay of each population. After application to the leaf disk, the acetone was allowed to evaporate leaving only the insecticide residue. Each insect was given the opportunity to consume the entire leaf disk over a 24‐h period and the percent mortality was recorded (*n* = 12–36 individual larvae per dose, depending on population). Assay plates were held at 26°C, 70% RH, and a 16:8 h light/dark photoperiod. A probit regression analysis was conducted (PROC PROBIT, SAS Institute, v. 9.4), and used to estimate the phenotypic response to an insecticide exposure for each field location independently. A reference laboratory population with no prior pesticide exposure was included as a baseline for this investigation.

### 2.3 Targeted RNA sequencing

Adult beetles from five populations, Maine (Aroostook‐1), Maine (Aroostook‐2), Wisconsin (Dane), Wisconsin (Hancock), and Oregon (Umatilla), were fed on untreated potato foliage (Russet Burbank) for 72 h upon arrival at University of Wisconsin‐Madison. A susceptible laboratory population maintained at the University of Wisconsin‐Madison, with no prior history of insecticide exposure, was used as a reference population. After 72 h, six healthy and actively feeding adults from each population were individually placed in sterile 1.5 ml microcentrifuge tubes and flash frozen in liquid nitrogen. Total RNA was extracted from each adult with TriZol (Life Technology). DNA contamination was removed with TurboDNase (Life Technology) and total RNA was purified through ethanol (EtOH) precipitation, air dried until no visible liquid was observed, and then suspended in 50 μl DNase/RNase‐free H_2_O. Quality and quantity of RNA was measured on a Nanodrop (Thermo Fisher Scientific). All RNA concentrations were equalized before input into the complementary DNA (cDNA) synthesis kit, and the subsequent cDNA was generated with a Super Script III kit (Thermo Fisher Scientific). A total of 100 ng of insect cDNA for each biological replication (*n* = 6) was submitted to Floodlight Genomics LLC for targeted RNA sequencing. Targets for the amplification were selected based on data from two previous publications (Clements *et al*.^9^, ^10^) (Table S1). Eight transcripts from Clements *et al*. (2016) were chosen based on their patterns of overexpression in insecticide‐resistant, field populations examined within the study.^9^ Additionally, 30 transcripts were chosen from Clements *et al*. (2018) based on their patterns of overexpression when induced by imidacloprid and chlorothalonil in a laboratory‐based study.^10^ Transcript sequence for each transcript of interest were BLASTed against the *L. decemlineata* reference genome to standardize annotation.^14^ If no match was found for the transcript in the reference genome, the previous annotation from the original manuscript^9^, ^10^ was retained. Sequence of each transcript of interest was provided to Floodlight Genomics for processing using their MonsterPlex workflow (https://floodlightgenomics.com/location/). MonsterPlex is an optimized Hi‐Plex approach^23^ and includes custom primer design to the specified transcripts of interest, polymerase chain reaction (PCR) amplification of each biological replicate in triplicate to minimize amplification and instrumental variation and sequencing of the targets using the Illumina HiSeq X platform, according to the manufacturer's directions (primer and amplicon sequences from Floodlight Genomics; Table S2). Floodlight Genomics delivered sample‐specific raw transcript sequence reads and all work was conducted at no cost as part of Floodlight Genomics, Educational and Research Outreach Program. Raw reads were aligned to annotated sequences using Geneious bioinformatic software, which provided a total number of reads per sequence of interest for examining transcript expression.

### 2.4 Transcript expression analysis

Transcript counts were standardized to total number of reads mapped per biological replication. Technical replicates were averaged and transcript expression for each transcript of interest was normalized to the reference gene (ribosomal protein 4) within each sample. The laboratory colony expression was used as a baseline for transcript expression to assess overall transcript expression between individual populations, and this was visualized using a heat map, hierarchical clustering, and principle components analyses conducted in JMP (SAS Institute, v. Pro 13). To determine whether transcripts were differentially expressed between geographic regions, analysis of variance ( ANOVA) with Tukey's post hoc analysis was conducted on the standardized transcript counts of each geographically distinct population, and a value of *P* ≤ 0.05 was considered statistically significant (Table S3). Mean transcript counts and standard error can be found in Table S4.

### 2.5 Quantitative PCR analysis

Transcript expression of two target genes (cytochrome P450 6 K1 isoform X1 and UDP‐glucuronosyltransferase), quantified using the MonsterPlex‐targeted RNA sequence methodology, were validated by a quantitative polymerase chain reaction (qPCR) using an aliquot of the cDNA submitted to Floodlight Genomics. Ribosomal protein 4 was used as a reference gene in the analysis. The qPCR were run on a CFX‐96 platform (Bio‐Rad Laboratories) with a master mix of Bullseye EverGreen (MIDSCI). The qPCR were conducted using the Pfaffl efficiency calibrated methodology; primer and primer efficiency^24^ can be found in Table S5. Triplicate reactions were run at 95°C for 10 min, followed by 95°C for 15 s, and 62°C for 60 s for a total of 40 cycles. Mean cycle threshold (Ct) values were collected for each biological replicate and fold change estimates between the insecticide susceptible and putative resistant, or unknown populations were calculated. A two‐tailed Student's *t*‐test was conducted to determine if transcript expression differed between treatment groups and a value of *P* ≤ 0.05 was considered statistically significant. A correlation regression was conducted to determine if the targeted RNA sequencing data was significantly different from, or similar to the qPCR obtained in JMP. A correlation of *P* ≤ 0.05 was used to determine if there was significant correlation.

## 3 RESULTS

### 3.1 Median lethal dose estimates for geographically distinct populations of *Leptinotarsa decemlineata*

Imidacloprid median lethal dose assays demonstrate differences in imidacloprid susceptibility in different geographic regions, and between populations within regions. Larvae from the conventionally managed Wisconsin (Hancock) and Maine (Aroostook‐2) populations both demonstrated high levels of imidacloprid resistance compared with the susceptible laboratory population. Insects from the conventionally managed Oregon (Umatilla) population and both of the organically managed Wisconsin (Dane) and Maine (Aroostook‐1) populations demonstrated susceptibility to imidacloprid. Wisconsin (Hancock) and Maine (Aroostock‐2) are both conventionally managed farms in regions of modest to high insecticide and fungicide input.^20^ The Oregon (Umatilla) population historically has received insecticidal inputs comparable with those used at the Wisconsin (Hancock) and Maine (Aroostook‐2) sites; however, the population‐level response to imidacloprid was not statistically different from the susceptible laboratory colony in this study (Table 1).

**TABLE 1:** Imidacloprid median lethal dose (LD_50_) estimates for second‐instar *Leptinotarsa decemlineata* larvae from populations collected in Maine, Wisconsin, Oregon, and a laboratory, susceptible colony

Site	Year	*n*	Dose (ng)	95% CI^a^	χ^2^ ^b^	*P*‐value	RR
Laboratory Colony	2019	288	1.04	0.5–1.5	20.28	<0.0001	1.00
Oregon (Umatilla)^c^	2019	192	0.7	0.4–1.1	43.64	<0.0001	0.67
Wisconsin (Dane)^d^	2019	192	2.26	1.04–4.44	42.93	<0.0001	2.17
Wisconsin (Hancock)^c^	2019	192	15.1	8.65–25.67	36.95	<0.0001	14.52
Maine (Aroostook‐1)^d^	2019	96	1.42	0.5–3.43	23.41	<0.0001	1.37
Maine (Aroostook‐2)^c^	2019	96	11.5	3.8–24.38	12.9	0.0003	11.06

RR resistant ration calculated using the Lab Colony as a reference.

^a^ 95% confidence interval (CI) estimates around LD_50_ estimates.

^b^ Chi‐square analysis effects of the Proc Probit regression.

^c^ Conventional standard pesticide input population.

^d^ Organic standard pesticide input population.

### 3.2 Hierarchical clustering and heat map analysis of transcripts implicated in insecticide resistance

**FIGURE 1:**
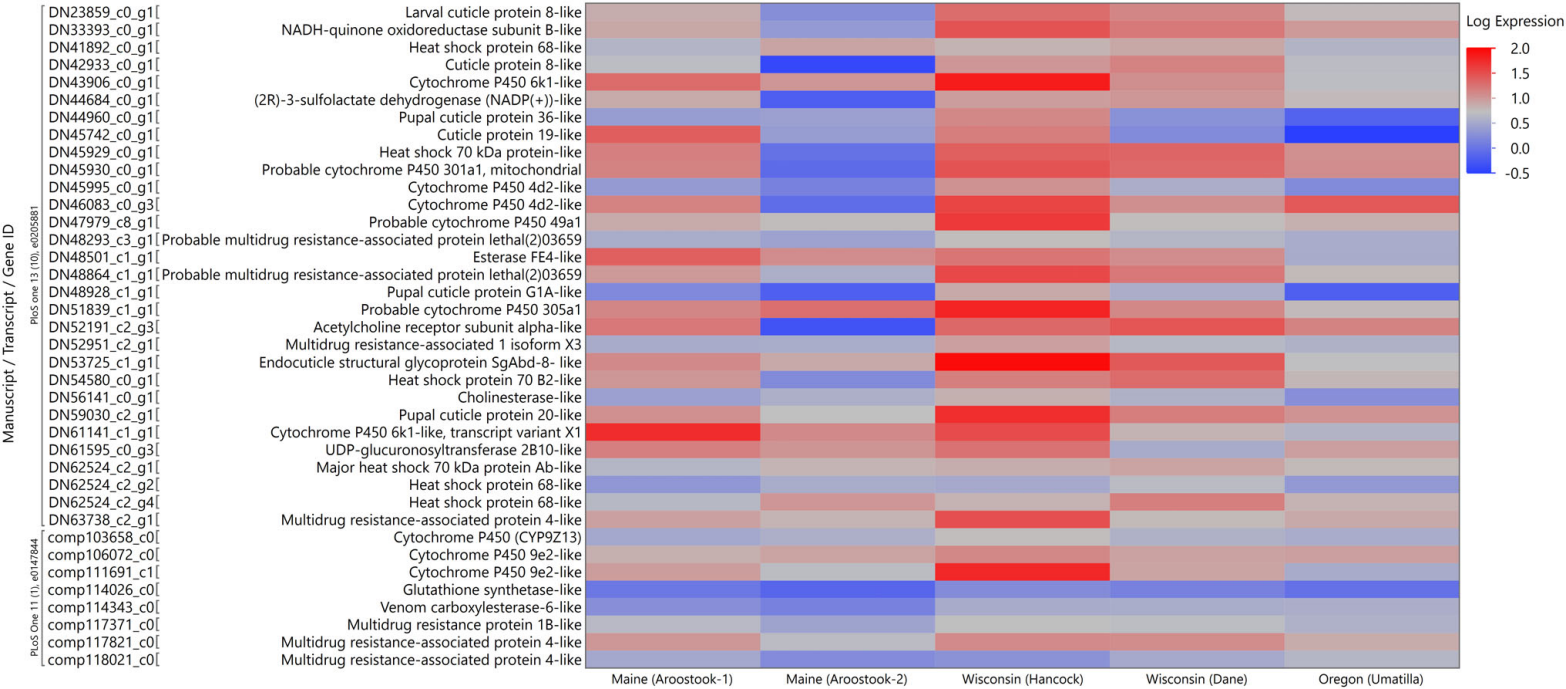
Heat map of log transcript expression values ranging from −0.5 to 2.0 of 38 transcripts previously associated with insecticide resistance across five *Leptinotarsa decemlineata* populations. Conventionally managed fields include Oregon (Umatilla), Wisconsin (Hancock), Maine (Aroostook‐2), while organically managed fields include Wisconsin (Dane) and Maine (Aroostook‐1).

Transcript expression of previously classified genes correlated to insecticide resistance were differentially expressed in different geographic regions in the current study. The conventional Wisconsin (Hancock) population of *L. decemlineata* overexpressed several transcripts that encode for possible xenobiotic detoxification mechanisms^9^, ^10^ (Figure 1). Specifically, the Wisconsin (Hancock) population overexpressed (greater than a log_2_ fold change) all but 4 of the 38 transcripts examined when compared with the susceptible laboratory population. The Wisconsin (Hancock) population did not overexpress glutathione synthetase‐like, multidrug resistance‐associated protein 4‐like, heat shock protein 68‐like, and venom carboxylesterase‐6‐like when compared with the susceptible laboratory population.

**FIGURE 2:**
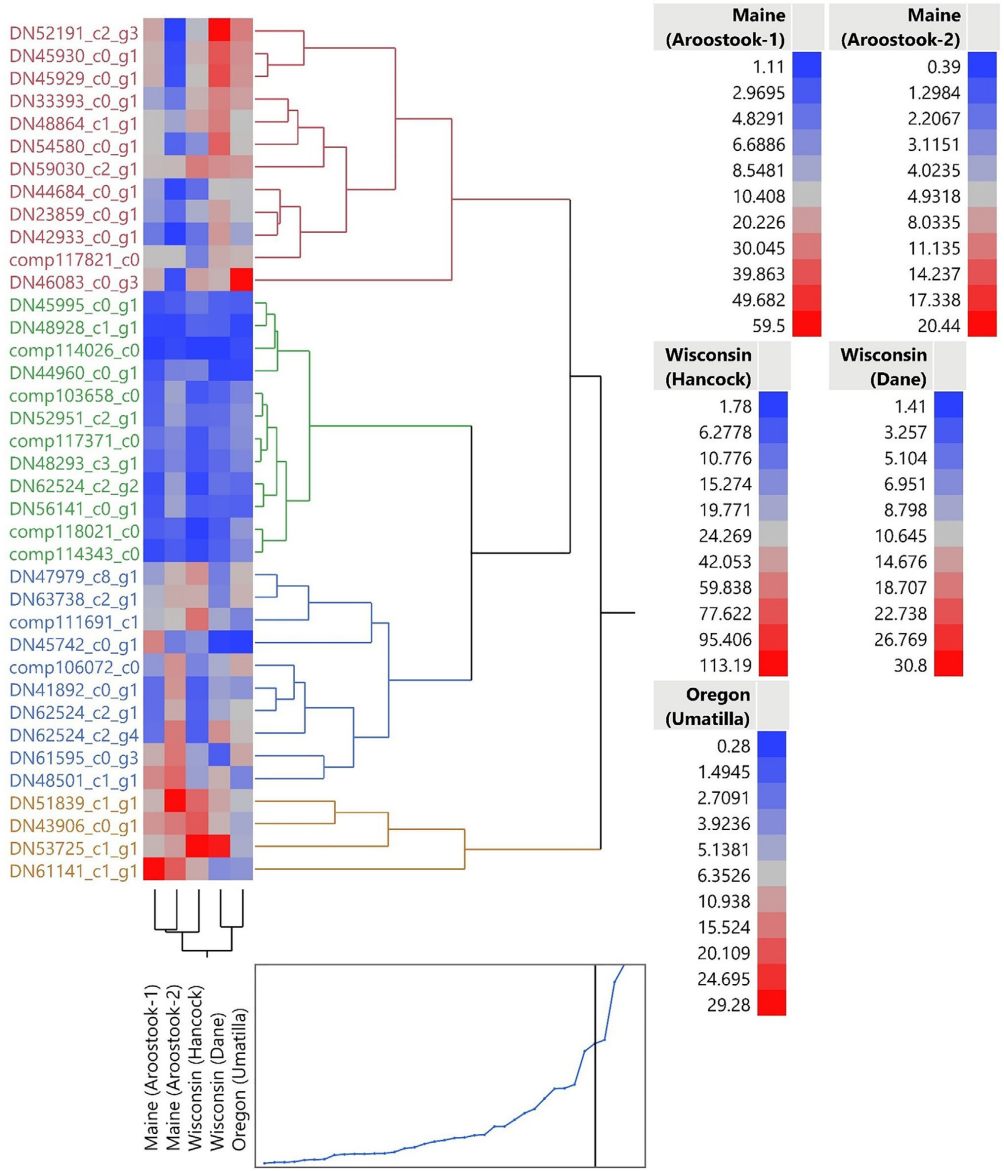
Standardized hierarchical clustering profiles of transcript expression among 38 previously described detoxification mechanisms comparing geographically discrete populations of *Leptinotarsa decemlineata*. Conventionally managed fields include Oregon (Umatilla), Wisconsin (Hancock), Maine (Aroostook‐2), while organically managed fields include Wisconsin (Dane) and Maine (Aroostook‐1).

A standardized hierarchical cluster analysis was conducted to compare insect populations, originating from different geographic regions, based on transcript expression (Figure 2). The standardized cluster analysis grouped geographic transcript expression of Maine (Aroostock‐1) and Maine (Aroostock‐2) together with a closely related side branch of Wisconsin (Hancock). It further grouped the Wisconsin (Dane) and Oregon (Umatilla) populations together (Figure 2). The standardized hierarchical clustering results were consistent with the results of a principle component analysis (Figure S1). This observation was solely based on a log_2_ fold change of raw reads which was visualized using both the heat map and hierarchical clustering analysis. To determine whether transcripts were differentially expressed between geographic regions, an ANOVA with Tukey's post hoc analysis was conducted on the standardized transcript counts of each geographically distinct population, and a value of *P* ≤ 0.05 was considered statistically significant (Table S3).

### 3.3 Transcript expression of documented detoxification mechanisms activated from pre‐exposure to imidacloprid and chlorothalonil

**FIGURE 3:**
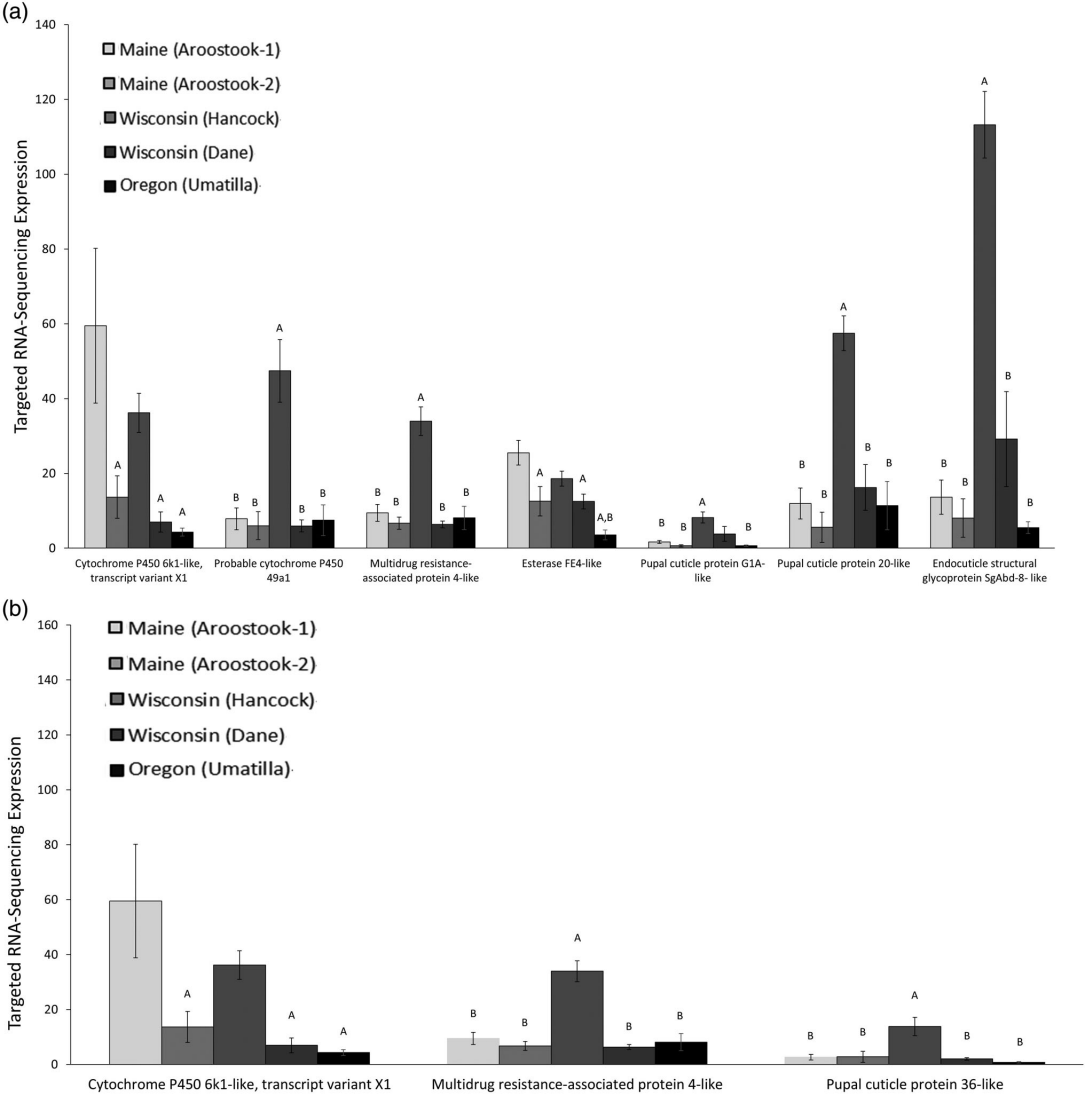
Constitutive expression of transcripts previously found to be upregulated in response to topical exposure to (a) imidacloprid or (b) chlorothalonil (from Clements *et al*.^10^) in five distinct field populations of *Leptinotarsa decemlineata*. Only transcripts that were significantly overexpressed are reported. Values represent mean ± SEM. Letters are used to denote significance differences for each gene. ‘A’ represents a significant difference (*P* < 0.05) when compared with Maine (Aroostook‐1) and ‘B’ represents a significant difference (*P* < 0.05) when compared with Wisconsin (Hancock). Conventionally managed fields include Oregon (Umatilla), Wisconsin (Hancock), Maine (Aroostook‐2), while organically managed fields include Wisconsin (Dane) and Maine (Aroostook‐1).

Targeted RNA sequencing revealed differential patterns of transcript overexpression among geographically separate populations. Transcripts examined were previously shown to be induced following exposure of *L. decemlineata* to either imidacloprid or chlorothalonil.^10^ From among the 13 transcripts identified as responsive to imidacloprid exposure in the earlier investigation, we noted a statistically significant change (*P* ≤ 0.05) in expression of seven of these transcripts in the Wisconsin (Hancock) and Maine (Aroostook‐2) populations (Figure 3a). From among the ten transcripts observed to be overexpressed in response to chlorothalonil in the same study, we observed three of these transcripts being overexpressed in the Wisconsin (Hancock) population (Figure 3b). These transcripts encode for multiple cytochrome P450s (cytochrome P450 6 k1‐like, transcript variant X1 and probable cytochrome P450 49a1), cuticular proteins (pupal cuticle protein 36‐like, pupal cuticle protein G1A‐like, endocuticle structural glycoprotein SgAbd‐8‐like, and pupal cuticle protein 20‐like), a multidrug resistant protein (multidrug resistance‐associated protein 4‐like) and an esterase (esterase FE4‐like). For this analysis, transcript expression was only compared between field locations (and not the lab population) to evaluate the difference in transcript expression within different agricultural field locations.

### 3.4 Quantitative PCR confirmation of targeted RNA sequencing data

**FIGURE 4:**
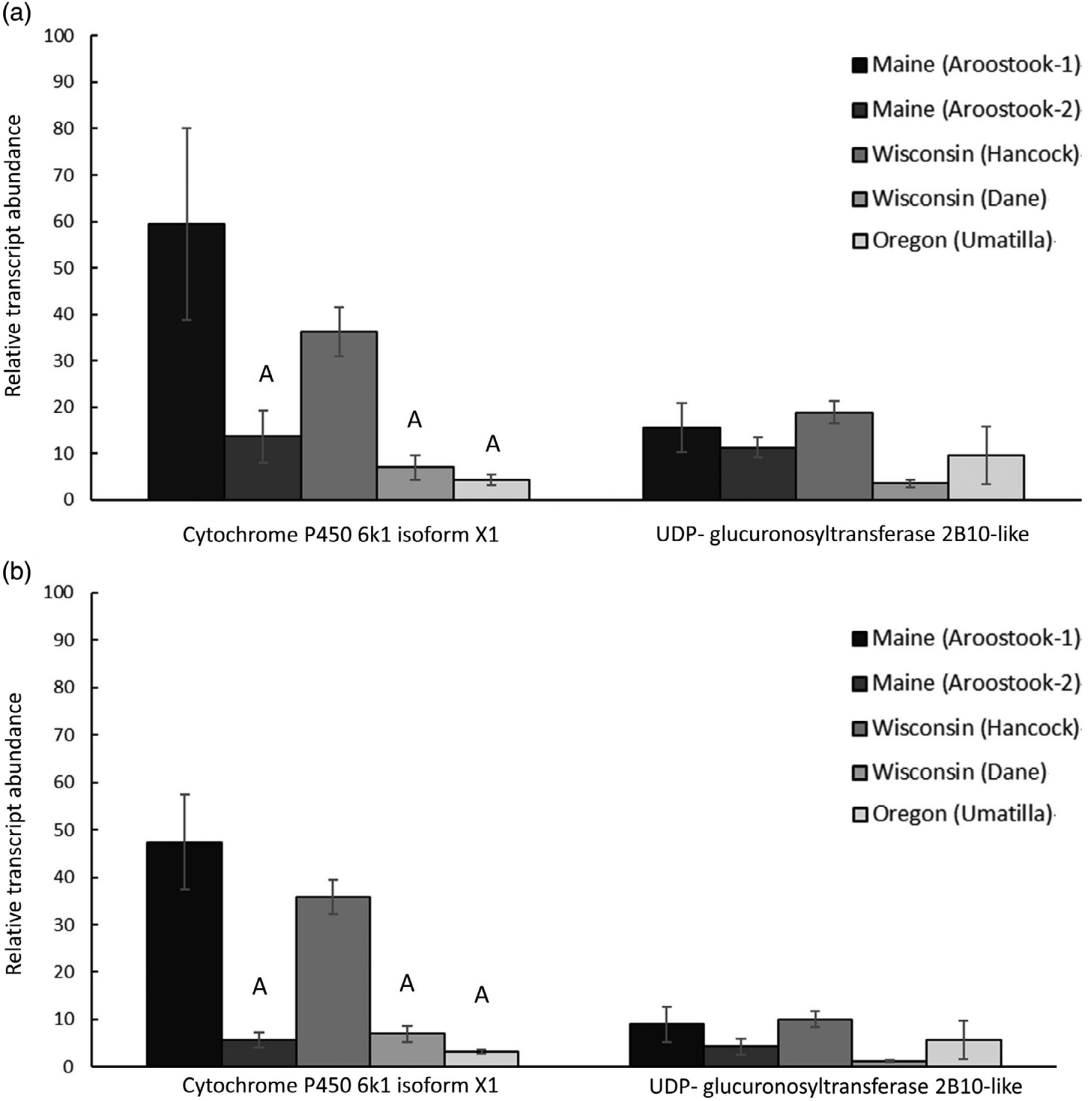
Quantitative polymerase chain reaction (qPCR) validation of targeted RNA sequencing of genes that encode for cytochrome p450 6 k1 isoform x1 and UDP‐glucuronosyltransferase 2B10‐like. (a) RNA sequencing fold change and (b) qPCR fold change. Values represent mean ± SEM and a significant correlation of *P* < 0.0001 was noted between qPCR and RNA sequencing. Letters are used to denote significance differences for each gene. ‘A’ represents a significant difference (*P* < 0.05) when compared with Maine (Aroostook‐1) and ‘B’ represents a significant difference (*P* < 0.05) when compared with Wisconsin (Hancock). Conventionally managed fields include Oregon (Umatilla), Wisconsin (Hancock), Maine (Aroostook‐2), while organically managed fields include Wisconsin (Dane) and Maine (Aroostook‐1).

Quantitative PCR confirmed the targeted RNA sequencing transcript expression results, with a highly significant correlation of *P* < 0.0001 (Figure 4). Quantitative PCR results of transcripts encoding protein production of cytochrome P450 6 K1 isoform X1 and UDP‐glucuronosyltransferase were statistically similar to the targeted RNA sequencing fold change calculations (Figure 4), validating the findings from the targeted RNA sequencing assay.

## 4 DISCUSSION AND CONCLUSION

*Leptinotarsa decemlineata* were collected from agroecosystems where potato production is prevalent; locations were selected based on observed variation in pesticide inputs (organic versus conventional management inputs) and variation in geographic location. The transcripts examined within this study have previously been correlated with xenobiotic detoxification (phase 1 and phase 2 enzymes), transport (multidrug resistant proteins), and reduced penetration (cuticular proteins).^9^, ^10^ The transcript profiles generated for each population were linked to the observed phenotypic resistance estimates (LD_50_ bioassays), and examined in the context of geographic location to determine patterns of insecticide resistance. Although we only examined a set of previously classified transcripts, other transcripts and associated resistance mechanisms should be examined in the future.

The estimates generated from the LD_50_ assays correspond with patterns of insecticide resistance among the different geographic regions of the USA. From among the five populations of insects examined, we determined that insects collected from fields exposed to conventional management inputs in the Midwest and on the East Coast had the highest estimated LD_50_ values. The populations were collected from the intensively managed Central Sands production region of Wisconsin (Hancock) and the Aroostook County production region in Maine (Aroostook‐2). Fields in these production regions have received at‐plant applications of carbamate and neonicotinoid insecticides for nearly 40 years. Historically, these field locations would also have been challenged with different mode of action pesticide groups, including fungicides and herbicides. By contrast, the two *L. decemlineata* populations collected from organically managed locations were highly susceptible to imidacloprid, similar to the susceptible laboratory population. The Oregon (Umatilla) population, which received conventional standard pesticide inputs, had estimated LD_50_ values that were statistically similar to the susceptible laboratory population. Although the insecticide regimen in the Pacific Northwest is generally similar to the Midwest and East Coast, on average, the Pacific Northwest receives only a fraction of the total fungicide applications.^20^ However, with only five fields examined in this study, we are unable to definitively identify how management inputs contribute to these trends in observed insensitivity. We conducted the LD_50_ studies using second‐instar larvae to ensure that insects were not exposed to any chemical inputs prior to the assay. Adult insects were used for the examination of transcript expression to standardize the life stage between the current study and results previously described in the Clements *et al*.'s 2016 and 2018 studies.^9^, ^10^

To understand the underlying mechanisms for resistance among geographically distinct populations, we examined transcript expression from among 38 transcripts previously implicated in insecticide resistance.^9^, ^10^ These transcripts were previously identified in an induction study with imidacloprid and chlorothalonil and by examining gene expression patterns between an imidacloprid resistant and susceptible field population.^9^, ^10^ Although the transcripts examined represent only a fraction of the classified enzymatic detoxification mechanisms in other insect taxa, we used these transcripts to investigate two primary questions. The first was to explore the similarities and differences in transcript expression of detoxification mechanisms between geographic regions. From our analysis, we noted that the population from Wisconsin (Hancock) overexpressed (greater than log_2_) all but 4 of the 38 transcripts examined (glutathione synthetase‐like, multidrug resistance‐associated protein 4‐like, heat shock protein 68‐like, and venom carboxylesterase‐6‐like) when compared with the susceptible laboratory population. This population was also found to have the highest LD_50_ values, suggesting that these overexpressed transcripts may aid *L. decemlineata* in coping with multiple pesticide selection pressures. A standardized hierarchical cluster analysis of beetle transcript expression profiles further indicated the populations from Maine grouped together, whereas the populations from Oregon (Umatilla) and Wisconsin (Dane) grouped together. The conventional population from Wisconsin (Hancock) was most closely associated with the Maine populations. Populations did not cluster together based on management practices (organic versus conventional) associated with these populations in the hierarchical analysis, potentially because molecular mechanisms of insecticide resistance have developed in different manners in the different geographic regions. One assumption within this study is that the transcripts observed are constitutively activated within a population. To assess constitutively active transcripts, insects collected from field locations were allowed to feed on untreated foliage for 72 h to allow transcript expression to be reoriented to baseline.^25^, ^26^

The second question examined in this study was to determine whether the transcripts noted as being induced by imidacloprid and chlorothalonil in laboratory investigations trials would be overexpressed in field populations. We confirmed that a large fraction of imidacloprid‐ and chlorothalonil‐induced transcripts were similarly overexpressed in field populations. To compare patterns of transcript expression among field locations, we did not include the susceptible laboratory population within the statistical analysis. Again, the Wisconsin (Hancock) population predominantly overexpressed resistance‐related transcripts more so than other field populations. Interestingly, the Maine (Aroostook‐1), organically managed population overexpressed transcripts related to cytochrome P450 6 k1‐like transcript variant X1 and esterase FE4‐like. The Maine (Aroostook‐1) population is managed with organic pesticide inputs. Many of the organic insecticides, including Entrust SC (spinosad), have target sites that are similar to those of non‐organically certified insecticides. It is possible that organic pesticides may be activating similar mechanisms of resistance. Within the imidacloprid induced transcript group, 7 of the 13 transcripts were overexpressed, and within the chlorothalonil induced group, three of ten transcripts were overexpressed. However, two of the transcripts overexpressed in the chlorothalonil group were shared with imidacloprid. These findings suggest that laboratory induction studies can be informative of the patterns of transcript expression observed within field‐collected *L. decemlineata* populations. Further, results reported here provide additional evidence for the induction of resistance mechanisms by both insecticide and fungicide applications. An interesting observation was that the two fields classified as imidacloprid‐resistant, Wisconsin (Hancock) and Maine (Aroostock‐2), had significantly different patterns of transcript expression when compared with each other, suggesting that the two populations may have independently developed insecticide resistance using different detoxification mechanisms even when exposed to similar stressors.

This investigation set out to determine whether geographic location and pest management practices could influence the patterns of resistance‐associated transcripts. By examining the transcript expression of multiple populations of *L. decemlineata*, we determined the similarities and dissimilarities between transcript expression of known xenobiotic detoxification mechanisms. By establishing the response of populations to insecticides through LD_50_ estimates and examining transcript expression through targeted RNA sequencing, we determined that the two populations deemed as imidacloprid‐resistant overexpress unique detoxification mechanisms in different proportions. We further validated the induction studies conducted by Clements *et al*. (2018) in field populations.^10^ The experiments conducted within this study demonstrate a diverse set of transcripts that appear to be overexpressed within different field populations with different resistant phenotypes. In this study, we did not examine whether the selected genes were responsible for the resistant phenotype. Further functional studies would be needed to validate that these genes are fully, or partially responsible for the insecticide resistance observed at these locations. The findings of this research further suggest that mechanisms of insecticide resistance in *L. decemlineata* most likely involve multiple mechanisms, and further that these mechanisms differ among geographic regions.

## ETHICS STATEMENT

United States Department of Agriculture Permit to Move Live Plant Pests, Noxious Weeds, and Soil P526‐180110‐008 was acquired for field collection of *L. decemlineata* for the study described.

## Supporting information


**Table S1.** Transcripts of interest examined during the targeted RNA sequencing study.

**Table S2.** MonsterPlex primers and amplicon.

**Table S3.** Statistical analysis of transcript expression between populations

**Table S4.** Mean transcript counts

**Table S5.** Quantitative PCR primers and primer efficiencies.

**Figure S1.** Principle component analysis of examined transcript expression by location

## Data Availability

All relevant data are contained within the article and its Supporting Information files.
