# Supplementary material for: Targeted RNA sequencing reveals differential patterns of transcript expression in geographically discrete, insecticide resistant populations of Leptinotarsa decemlineata
Source: Pest Manag Sci. 2021 May 3;77(7):3436–44. doi: 10.1002/ps.6393 (PMC8252485; doi:10.1002/ps.6393)
Supplement: Supplementary file 1 — Table S1. Transcripts of interest examined during the targeted RNA sequencing study. [file PS-77-3436-s005.docx]

**Supplemental Table S1:** Transcripts of interest examined during the targeted RNA sequencing study.

| **Manuscript** | **Transcript ID** | **Sequence Length** | **Insect taxa** | **NCBI Gene Designation** | **NCBI Gene ID** |
| --- | --- | --- | --- | --- | --- |
| PLoS One 11 (1), e0147844 | comp103658_c0 | 755 | *Leptinotarsa decemlineata* | Cytochrome P450 (CYP9Z13) | (DQ631659.1) |
| PLoS One 11 (1), e0147844 | comp106072_c0 | 1084 | *Leptinotarsa decemlineata* | Cytochrome P450 9e2-like | (LOC111508872) |
| PLoS One 11 (1), e0147844 | comp111691_c1 | 2124 | *Leptinotarsa decemlineata* | Cytochrome P450 9e2-like | (LOC111507099) |
| PLoS One 11 (1), e0147844 | comp114026_c0 | 2118 | *Leptinotarsa decemlineata* | Glutathione synthetase-like | (LOC111502309) |
| PLoS One 11 (1), e0147844 | comp114343_c0 | 358 | *Leptinotarsa decemlineata* | Venom carboxylesterase-6-like | (LOC111507303) |
| PLoS One 11 (1), e0147844 | comp117371_c0 | 2743 | *Leptinotarsa decemlineata* | Multidrug resistance protein 1B-like | (LOC111510069) |
| PLoS One 11 (1), e0147844 | comp117821_c0 | 3276 | *Leptinotarsa decemlineata* | Multidrug resistance-associated protein 4-like | (LOC111512693) |
| PLoS One 11 (1), e0147844 | comp118021_c0 | 4430 | *Leptinotarsa decemlineata* | Multidrug resistance-associated protein 4-like | (LOC111503007) |
| PloS one 13 (10), e0205881 | DN23859_c0_g1 | 669 | *Leptinotarsa decemlineata* | Larval cuticle protein 8-like | (LOC111515168) |
| PloS one 13 (10), e0205881 | DN33393_c0_g1 | 863 | *Leptinotarsa decemlineata* | NADH-quinone oxidoreductase subunit B-like | (LOC111510228) |
| PloS one 13 (10), e0205881 | DN41892_c0_g1 | 228 | *Leptinotarsa decemlineata* | Heat shock protein 68-like | (LOC111515343) |
| PloS one 13 (10), e0205881 | DN42933_c0_g1 | 837 | *Leptinotarsa decemlineata* | Cuticle protein 8-like | (LOC111508375) |
| PloS one 13 (10), e0205881 | DN43906_c0_g1 | 1897 | *Leptinotarsa decemlineata* | Cytochrome P450 6k1-like | (LOC111505524) |
| PloS one 13 (10), e0205881 | DN44684_c0_g1 | 1686 | *Leptinotarsa decemlineata* | (2R)-3-sulfolactate dehydrogenase (NADP(+))-like | (LOC111506923) |
| PloS one 13 (10), e0205881 | DN44960_c0_g1 | 1055 | *Leptinotarsa decemlineata* | Pupal cuticle protein 36-like | (LOC111515989) |
| PloS one 13 (10), e0205881 | DN45742_c0_g1 | 1110 | *Leptinotarsa decemlineata* | Cuticle protein 19-like | (LOC111505464) |
| PloS one 13 (10), e0205881 | DN45929_c0_g1 | 2246 | *Leptinotarsa decemlineata* | Heat shock 70 kDa protein-like | (LOC111509239) |
| PloS one 13 (10), e0205881 | DN45930_c0_g1 | 1925 | *Leptinotarsa decemlineata* | Probable cytochrome P450 301a1, mitochondrial | (LOC111508632) |
| PloS one 13 (10), e0205881 | DN45995_c0_g1 | 1520 | *Leptinotarsa decemlineata* | Cytochrome P450 4d2-like | (LOC111502951) |
| PloS one 13 (10), e0205881 | DN46083_c0_g3 | 1621 | *Leptinotarsa decemlineata* | Cytochrome P450 4d2-like | (LOC111505305) |
| PloS one 13 (10), e0205881 | DN47979_c8_g1 | 1853 | *Leptinotarsa decemlineata* | Probable cytochrome P450 49a1 | (LOC111518140) |
| PloS one 13 (10), e0205881 | DN48293_c3_g1 | 2438 | *Leptinotarsa decemlineata* | Probable multidrug resistance-associated protein lethal(2)03659 | (LOC111511378) |
| PloS one 13 (10), e0205881 | DN48501_c1_g1 | 992 | *Leptinotarsa decemlineata* | Esterase FE4-like | (LOC111509267) |
| PloS one 13 (10), e0205881 | DN48864_c1_g1 | 4549 | *Leptinotarsa decemlineata* | Probable multidrug resistance-associated protein lethal(2)03659 | (LOC111505667) |
| PloS one 13 (10), e0205881 | DN48928_c1_g1 | 1719 | *Leptinotarsa decemlineata* | Pupal cuticle protein G1A-like | (LOC111506222) |
| PloS one 13 (10), e0205881 | DN51839_c1_g1 | 2320 | *Leptinotarsa decemlineata* | Probable cytochrome P450 305a1 | (LOC111504977) |
| PloS one 13 (10), e0205881 | DN52191_c2_g3 | 790 | *Anoplophora glabripennis* | Acetylcholine receptor subunit alpha-like | (XP_018577209.1) |
| PloS one 13 (10), e0205881 | DN52951_c2_g1 | 750 | *Anoplophora glabripennis* | Multidrug resistance-associated 1 isoform X3 | (XP_018568572.1) |
| PloS one 13 (10), e0205881 | DN53725_c1_g1 | 2078 | *Anoplophora glabripennis* | Endocuticle structural glycoprotein SgAbd-8- like | (XP_018578131.1) |
| PloS one 13 (10), e0205881 | DN54580_c0_g1 | 1603 | *Leptinotarsa decemlineata* | Heat shock protein 70 B2-like | (LOC111510567) |
| PloS one 13 (10), e0205881 | DN56141_c0_g1 | 1729 | *Leptinotarsa decemlineata* | Cholinesterase-like | (LOC111505998) |
| PloS one 13 (10), e0205881 | DN59030_c2_g1 | 1018 | *Aethina tumida* | Pupal cuticle protein 20-like | (XP_019867111.1) |
| PloS one 13 (10), e0205881 | DN61141_c1_g1 | 1879 | *Leptinotarsa decemlineata* | Cytochrome P450 6k1-like, transcript variant X1 | (LOC111504268) |
| PloS one 13 (10), e0205881 | DN61595_c0_g3 | 2250 | *Leptinotarsa decemlineata* | UDP-glucuronosyltransferase 2B10-like | (LOC111511174) |
| PloS one 13 (10), e0205881 | DN62524_c2_g1 | 1282 | *Leptinotarsa decemlineata* | Major heat shock 70 kDa protein Ab-like | (LOC111515127) |
| PloS one 13 (10), e0205881 | DN62524_c2_g2 | 657 | *Leptinotarsa decemlineata* | Heat shock protein 68-like | (LOC111515390) |
| PloS one 13 (10), e0205881 | DN62524_c2_g4 | 207 | *Leptinotarsa decemlineata* | Heat shock protein 68-like | (LOC111515343) |
| PloS one 13 (10), e0205881 | DN63738_c2_g1 | 931 | *Leptinotarsa decemlineata* | Multidrug resistance-associated protein 4-like | (LOC111507851) |
| Ref Gene | RP18 | 367 | *Leptinotarsa decemlineata* | 60S ribosomal protein L18 | (LOC111516834) |
| Ref Gene | RP4 | 455 | *Leptinotarsa decemlineata* | 60S ribosomal protein L4 | (LOC111509998) |
| Ref Gene | ARF1 | 642 | *Leptinotarsa decemlineata* | ADP-ribosylation factor-like protein 1 | (LOC111513661) |
| Ref Gene | ARF4 | 1128 | *Leptinotarsa decemlineata* | ADP-ribosylation factor-like protein 4C, transcript variant X5 | (LOC111512786) |

*Transcript designation was standardized to genomic assembles from Schoville et al. 2018 [14]
