# Supplementary material for: Targeted RNA sequencing reveals differential patterns of transcript expression in geographically discrete, insecticide resistant populations of Leptinotarsa decemlineata
Source: Pest Manag Sci. 2021 May 3;77(7):3436–44. doi: 10.1002/ps.6393 (PMC8252485; doi:10.1002/ps.6393)
Supplement: Supplementary file 2 — Table S2. MonsterPlex primers and amplicon. [file PS-77-3436-s003.docx]

**Supplemental Table S2:** MonsterPlex primers and amplicon.

| **Seq ID** | **Forward** | **Reverse** | **MonsterPlex amplicon** |
| --- | --- | --- | --- |
| comp103658_c0 | GCCGAAGGATTGATATTGCCTTT | GACCCACTGCGTTTCGATCC | GCCGAAGGATTGATATTGCCTTTATTTTCCTCACTGAATCTTTCTGGATCGAAACGCAGTGGGTC |
| comp106072_c0 | CGCATTTTGCTGCTGGTGAA | ACCGGTCAGAAATGGCGAGA | CGCATTTTGCTGCTGGTGAAGGATGGACTGAGAATAGGCCGCATATCTCGCCATTTCTGACCGGT |
| comp111691_c1 | CGAAACTGGCAGCCGGAATA | CACGCCATACGGAGCTGATG | CGAAACTGGCAGCCGGAATATCAAGATGGCGGTCGGTTTTTAGAGCTCATCAGCTCCGTATGGCGTG |
| comp114026_c0 | CCATGCAAGATCGCCCAGTC | TGCCTTTGCCAGACCAACAA | CCATGCAAGATCGCCCAGTCTCTAGTTTTCCTCACCAAGTCTTTCAGTTGTTGGTCTGGCAAAGGCA |
| comp114343_c0 | CACTGGGAAAACAACAGCCATTC | TTCCAATGGGAGGAACTGCAT | CACTGGGAAAACAACAGCCATTCTTTGCTTACAAGGGAATACCGTATGCAGTTCCTCCCATTGGAA |
| comp117371_c0 | CCCACTCCGTTGGCTTTGTC | AATGTTTCGGGCCATGTTGG | CCCACTCCGTTGGCTTTGTCGTCAAAAAATCCAATTTCCTGGACCAACATGGCCCGAAACATT |
| comp117821_c0 | TGCCAGGTGTCCAGTCCAAA | TCGCATCGAATCTGGCATTTT | TGCCAGGTGTCCAGTCCAAATGTAAGTCATCAGAAGGAGATAAAATGCCAGATTCGATGCGA |
| comp118021_c0 | GGCGTGGTTCAGTTTGAGCA | TAAGGATTCGGGTCGCTTGC | GGCGTGGTTCAGTTTGAGCATTTTCCTGTAGATAACTGAGCAGCAAGCGACCCGAATCCTTA |
| DN23859_c0_g1 | CATCGGCTTTCCATCACCTG | CCTGCCAACGAAGCAATCG | CATCGGCTTTCCATCACCTGATGAAATAGTTTCACAGATTAGTTCCGGAGCGATTGCTTCGTTGGCAGG |
| DN33393_c0_g1 | TTTGTGGCCCATGACATTCG | GGAGCCGCAAATTGCATCAT | TTTGTGGCCCATGACATTCGGACTGGCATGTTGTGCTGTTGAAATGATGCAATTTGCGGCTCC |
| DN41892_c0_g1 | TGAAGGAAACTGCAGAGGCGTA | TGGCACTGTTATAACGGCATCTC | TGAAGGAAACTGCAGAGGCGTATTTGGGAAGTTCGATCAGAGATGCCGTTATAACAGTGCCA |
| DN42933_c0_g1 | TCTGCTAACATCGTTGCCCAAA | TGGGTGGGCATATTCTTCTTCG | TCTGCTAACATCGTTGCCCAAACGACTTTTTCCAAAGGACACGAAGAAGAATATGCCCACCCA |
| DN43906_c0_g1 | GCCTGCCCAATGCATGATAA | CTTCTCCACACCGGCGTCTT | GCCTGCCCAATGCATGATAACATACAGTCGAATATGCTTTTTTTTCAAAAGACGCCGGTGTGGAGAAG |
| DN44684_c0_g1 | TTGCCCCAGGCTTTGAACAT | GGGTCAGCCGGTTCCATTTT | TTGCCCCAGGCTTTGAACATCGCTTAAGTGACCTTCTGAATTCGATAAGGAAAATGGAACCGGCTGACCC |
| DN44960_c0_g1 | AGGCTACGGAAGGGGACCAG | GCACCTCCCTGTCCTCCAAA | AGGCTACGGAAGGGGACCAGCAGGTAGCGCTGGAGGACCAGGTTTTGGAGGACAGGGAGGTGC |
| DN45742_c0_g1 | GGGCACCGCTGCTGTAGACT | TGCGGCATCGAACTCCTTATC | GGGCACCGCTGCTGTAGACTGAGATAATGGCAGTTTCTGAGATAAGGAGTTCGATGCCGCA |
| DN45929_c0_g1 | GATTGCCGATGCCGAGAAGT | GCAGCCACAGCAGCTTTCAC | GATTGCCGATGCCGAGAAGTTCAAAGAGCATGATCTAGAAGTGAAAGCTGCTGTGGCTGC |
| DN45930_c0_g1 | GATGAACTTGGCGGCTTTGG | ATGTCCGAACGGCATGGAAG | GATGAACTTGGCGGCTTTGGAAATGAATATCAGGCGTTTGCTTCCATGCCGTTCGGACAT |
| DN45995_c0_g1 | TGGTGGACATGGCTCTACGG | GATGGTGCGCCAATGCATAC | TGGTGGACATGGCTCTACGGCTACTGGTTTAGCCTTCACCCTGTATGCATTGGCGCACCATC |
| DN46083_c0_g3 | ACATTGCCGGAGCGACATCT | ATCGTGGTTTCGGCCTTCCT | ACATTGCCGGAGCGACATCTTTTGGGTATGACTTTGGAAGTCAGGAAGGCCGAAACCACGAT |
| DN47979_c8_g1 | GTTCGTGTTCGACGGTGACG | TGAGGCAGGGTTTCCTCTCG | GTTCGTGTTCGACGGTGACGATATCAAGAAGGTATTCAAACGAGAGGAAACCCTGCCTCA |
| DN48293_c3_g1 | ACGGAATCGGACGGGAAAAT | TCGTGGCCCATGAGTTCAGA | ACGGAATCGGACGGGAAAATAGTCGCTGCGCATGTTACGAATTCTGAACTCATGGGCCACGA |
| DN48501_c1_g1 | CAGCCTGTTGACAAATGGGAAG | CGGGCACAGTGGACCATCTT | CAGCCTGTTGACAAATGGGAAGGAGTATACGATGCGACCAAAGATGGTCCACTGTGCCCG |
| DN48864_c1_g1 | CGCAGGTTCCGTGAGACAGA | CCCTGTAGCGGAGCAGTTCG | CGCAGGTTCCGTGAGACAGAACATCCTTTTCGGCAGGGAATTCGAACTGCTCCGCTACAGGG |
| DN48928_c1_g1 | ATGCGCTGTAAGCGGGAAGA | GCCCCCGTAGTATCCGCCTA | ATGCGCTGTAAGCGGGAAGAGCATAGGCACCGTGAGCGGCATAGGCGGATACTACGGGGGC |
| DN51839_c1_g1 | TCAAAGCGATGTTGATGGAACC | CGTCTCGCCCTTCTGTCCAT | TCAAAGCGATGTTGATGGAACCTATTCGAGAACATTTTGAAACATGGACAGAAGGGCGAGACG |
| DN52191_c2_g3 | GAGCTCCTCTGGAATGGCAAA | TCGGATTTGCTCGGTTGGAT | GAGCTCCTCTGGAATGGCAAATTATTCAATTCAGCCGTCGGCAGTGCCATCCAACCGAGCAAATCCGA |
| DN52951_c2_g1 | AAAAATGACATGGGGCATCG | GGCTGCCAGTGTGACTTTGC | AAAAATGACATGGGGCATCGTAAGATACCTTTGAGCAAATTCTTATGCAAAGTCACACTGGCAGCC |
| DN53725_c1_g1 | TCCCGTCATGTTTCCGTCCT | CCAGTGTGCGTTGTGTTCTCG | TCCCGTCATGTTTCCGTCCTGTACTGTCCTGTCACAGTGAGCCGAGAACACAACGCACACTGG |
| DN54580_c0_g1 | AGGAAACCGGACGACCCCTA | GCAGCATCCCCGATGAGTCT | AGGAAACCGGACGACCCCTAGCTATGTGGCTTTTACCGAAACTGAAAGACTCATCGGGGATGCTGC |
| DN56141_c0_g1 | CCTGCGACCGCAGACCTACT | TTTCTGCCTCGGCGTTTCTC | CCTGCGACCGCAGACCTACTTCAAAACTACTAGAGTGTCTGAGAAACGCCGAGGCAGAAA |
| DN59030_c2_g1 | GGGTTCCAAGCATCACCAGTT | TGAAGCAGCTACTGGCCTGGT | GGGTTCCAAGCATCACCAGTTAGTGCCAACAAACCAACACTAACCAGGCCAGTAGCTGCTTCA |
| DN61141_c1_g1 | GGTGAACGCTAGGCATGAAGC | ATGCCAAAAGCGGTGGATGT | GGTGAACGCTAGGCATGAAGCAGCCCTCTATGGGGCCGAGGTTATAACATCCACCGCTTTTGGCAT |
| DN61595_c0_g3 | GGGAAATCCGCAACCTCCAT | AGCGTCATTTGGCCGTGGTA | GGGAAATCCGCAACCTCCATCTTTATACTCAGAAATGTTTCTTCCTTACCACGGCCAAATGACGCT |
| DN62524_c2_g1 | TTGAACGACGCCAAAATGGA | GGTGGATCCTCCGACAAGGA | TTGAACGACGCCAAAATGGACAAGGGACAGATTCATGATGTAGTCCTTGTCGGAGGATCCACC |
| DN62524_c2_g2 | GGGGCCAACAAAACAACCAG | CCACAACTTCCAGGCATTCCA | GGGGCCAACAAAACAACCAGTATGGAGGAAATGCATCCAATGGAATGCCTGGAAGTTGTGG |
| DN62524_c2_g4 | TGAACGCAACGCCAGGATT | TGCGGGTTGATTGTCTGCAT | TGAACGCAACGCCAGGATTCCATGCAAGCAATCGCAGACCTTCACCACCTATGCAGACAATCAACCCGCA |
| DN63738_c2_g1 | AGAGCAGGAGGTGGCTGGTG | TCTGACCAATCGTGTATCCCAAAA | AGAGCAGGAGGTGGCTGGTGCAGAATATTCATTCTACTTTTGGGATACACGATTGGTCAGA |
