# Supplementary material for: Targeted RNA sequencing reveals differential patterns of transcript expression in geographically discrete, insecticide resistant populations of Leptinotarsa decemlineata
Source: Pest Manag Sci. 2021 May 3;77(7):3436–44. doi: 10.1002/ps.6393 (PMC8252485; doi:10.1002/ps.6393)
Supplement: Supplementary file 3 — Table S3. Statistical analysis of transcript expression between populations [file PS-77-3436-s001.docx]

**Supplemental Table S3.** Statistical analysis of transcript expression between populations. P values represent results of an ANOVA with Tukey’s post hoc analysis. A p < 0.05 was considered statistically significant.

| Compassion Transcript ID | | | | | | |
| --- | --- | --- | --- | --- | --- | --- |
|  | **comp103658_c0** | **comp106072_c0** | **comp111691_c1** | **comp114026_c0** | **comp114343_c0** | **comp117371_c0** |
| **Wisconsin (Hancock) vs Oregon** | 0.11 | 0.50 | <0.01 | 0.03 | 1.00 | 0.63 |
| **Wisconsin (Hancock) vs Wisconsin (Dane)** | 0.27 | 0.43 | <0.01 | 0.58 | 1.00 | 1.00 |
| **Wisconsin (Hancock) vs Maine (Arostook-2)** | 0.25 | 0.42 | <0.01 | 0.09 | 0.18 | 0.23 |
| **Wisconsin (Hancock) vs Maine (Arostook-1)** | 0.06 | 0.15 | <0.01 | <0.01 | 0.39 | 0.93 |
| **Maine (Arostook-1) vs Oregon** | 1.00 | 0.92 | 0.96 | 0.97 | 0.21 | 0.97 |
| **Maine (Arostook-1) vs Wisconsin (Dane)** | 0.91 | 0.95 | 1.00 | 0.74 | 0.23 | 0.99 |
| **Maine (Arostook-1) vs Maine (Arostook-2)** | 0.96 | 0.98 | 0.99 | 0.66 | 0.98 | 0.60 |
| **Maine (Arostook-2) vs Oregon** | 1.00 | 1.00 | 1.00 | 0.94 | 0.09 | 0.91 |
| **Maine (Arostook-2) vs Wisconsin (Dane)** | 1.00 | 1.00 | 0.99 | 0.12 | 0.10 | 0.34 |
| **Wisconsin (Dane) vs Oregon** | 0.98 | 1.00 | 0.97 | 0.37 | 1.00 | 0.81 |
|  |  |  |  |  |  |  |
|  | **comp117821_c0** | **comp118021_c0** | **DN23859_c0_g1** | **DN33393_c0_g1** | **DN41892_c0_g1** | **DN42933_c0_g1** |
| **Wisconsin (Hancock) vs Oregon** | 0.30 | 0.50 | 0.38 | 0.13 | 0.75 | 0.85 |
| **Wisconsin (Hancock) vs Wisconsin (Dane)** | 1.00 | 0.88 | 0.92 | 0.48 | 0.98 | 0.95 |
| **Wisconsin (Hancock) vs Maine (Arostook-2)** | 0.07 | 1.00 | 0.20 | 0.02 | 0.95 | 0.45 |
| **Wisconsin (Hancock) vs Maine (Arostook-1)** | 0.89 | 0.91 | 0.49 | 0.08 | 0.78 | 0.87 |
| **Maine (Arostook-1) vs Oregon** | 0.80 | 0.92 | 1.00 | 1.00 | 1.00 | 1.00 |
| **Maine (Arostook-1) vs Wisconsin (Dane)** | 0.91 | 1.00 | 0.91 | 0.79 | 0.39 | 0.43 |
| **Maine (Arostook-1) vs Maine (Arostook-2)** | 0.28 | 0.83 | 0.95 | 0.95 | 0.34 | 0.92 |
| **Maine (Arostook-2) vs Oregon** | 0.87 | 0.38 | 0.98 | 0.88 | 0.31 | 0.93 |
| **Maine (Arostook-2) vs Wisconsin (Dane)** | 0.06 | 0.79 | 0.56 | 0.41 | 1.00 | 0.13 |
| **Wisconsin (Dane) vs Oregon** | 0.31 | 0.95 | 0.83 | 0.90 | 0.36 | 0.40 |
|  |  |  |  |  |  |  |
|  | **DN43906_c0_g1** | **DN44684_c0_g1** | **DN44960_c0_g1** | **DN45742_c0_g1** | **DN45929_c0_g1** | **DN45930_c0_g1** |
| **Wisconsin (Hancock) vs Oregon** | <0.01 | 0.96 | <0.01 | 0.86 | 0.76 | 0.45 |
| **Wisconsin (Hancock) vs Wisconsin (Dane)** | <0.01 | 1.00 | <0.01 | 0.90 | 1.00 | 0.92 |
| **Wisconsin (Hancock) vs Maine (Arostook-2)** | <0.01 | 0.52 | <0.01 | 0.93 | 0.27 | 0.10 |
| **Wisconsin (Hancock) vs Maine (Arostook-1)** | <0.01 | 1.00 | <0.01 | 0.96 | 0.90 | 0.56 |
| **Maine (Arostook-1) vs Oregon** | 0.33 | 1.00 | 0.91 | 0.44 | 1.00 | 1.00 |
| **Maine (Arostook-1) vs Wisconsin (Dane)** | 0.79 | 0.98 | 1.00 | 0.49 | 0.94 | 0.95 |
| **Maine (Arostook-1) vs Maine (Arostook-2)** | 0.75 | 0.69 | 1.00 | 0.57 | 0.71 | 0.73 |
| **Maine (Arostook-2) vs Oregon** | 0.97 | 0.86 | 0.90 | 1.00 | 0.87 | 0.83 |
| **Maine (Arostook-2) vs Wisconsin (Dane)** | 1.00 | 0.39 | 1.00 | 1.00 | 0.30 | 0.34 |
| **Wisconsin (Dane) vs Oregon** | 0.93 | 0.91 | 0.98 | 1.00 | 0.82 | 0.89 |
|  |  |  |  |  |  |  |
|  | **DN45995_c0_g1** | **DN46083_c0_g3** | **DN47979_c8_g1** | **DN48293_c3_g1** | **DN48501_c1_g1** | **DN48864_c1_g1** |
| **Wisconsin (Hancock) vs Oregon** | <0.01 | 0.98 | <0.01 | 0.37 | <0.01 | <0.01 |
| **Wisconsin (Hancock) vs Wisconsin (Dane)** | <0.01 | 0.62 | <0.01 | 0.77 | 0.50 | 0.06 |
| **Wisconsin (Hancock) vs Maine (Arostook-2)** | <0.01 | 0.71 | <0.01 | 0.14 | 0.55 | <0.01 |
| **Wisconsin (Hancock) vs Maine (Arostook-1)** | <0.01 | 0.33 | <0.01 | 0.40 | 0.38 | <0.01 |
| **Maine (Arostook-1) vs Oregon** | 0.99 | 0.59 | 1.00 | 1.00 | <0.01 | 0.97 |
| **Maine (Arostook-1) vs Wisconsin (Dane)** | 0.87 | 0.97 | 1.00 | 0.96 | 0.01 | 0.80 |
| **Maine (Arostook-1) vs Maine (Arostook-2)** | 0.96 | 0.94 | 1.00 | 0.94 | 0.02 | 0.87 |
| **Maine (Arostook-2) vs Oregon** | 1.00 | 0.94 | 1.00 | 0.96 | 0.15 | 1.00 |
| **Maine (Arostook-2) vs Wisconsin (Dane)** | 0.52 | 1.00 | 1.00 | 0.64 | 1.00 | 0.29 |
| **Wisconsin (Dane) vs Oregon** | 0.60 | 0.88 | 1.00 | 0.95 | 0.13 | 0.43 |
|  |  |  |  |  |  |  |
|  | **DN48928_c1_g1** | **DN51839_c1_g1** | **DN52191_c2_g3** | **DN52951_c2_g1** | **DN53725_c1_g1** | **DN54580_c0_g1** |
| **Wisconsin (Hancock) vs Oregon** | <0.01 | <0.01 | 0.99 | 0.03 | <0.01 | 0.84 |
| **Wisconsin (Hancock) vs Wisconsin (Dane)** | 0.09 | <0.01 | 0.99 | 0.07 | <0.01 | 0.97 |
| **Wisconsin (Hancock) vs Maine (Arostook-2)** | <0.01 | <0.01 | 0.65 | 0.03 | <0.01 | 0.56 |
| **Wisconsin (Hancock) vs Maine (Arostook-1)** | <0.01 | <0.01 | 1.00 | 0.01 | <0.01 | 0.98 |
| **Maine (Arostook-1) vs Oregon** | 0.96 | 0.95 | 1.00 | 1.00 | 0.94 | 0.99 |
| **Maine (Arostook-1) vs Wisconsin (Dane)** | 0.65 | 1.00 | 0.91 | 0.95 | 0.59 | 0.73 |
| **Maine (Arostook-1) vs Maine (Arostook-2)** | 0.97 | 0.98 | 0.79 | 1.00 | 0.99 | 0.84 |
| **Maine (Arostook-2) vs Oregon** | 1.00 | 0.75 | 0.88 | 1.00 | 1.00 | 0.98 |
| **Maine (Arostook-2) vs Wisconsin (Dane)** | 0.33 | 0.98 | 0.32 | 0.98 | 0.34 | 0.21 |
| **Wisconsin (Dane) vs Oregon** | 0.28 | 0.96 | 0.82 | 0.99 | 0.20 | 0.44 |
|  |  |  |  |  |  |  |
|  | **DN56141_c0_g1** | **DN59030_c2_g1** | **DN61141_c1_g1** | **DN61595_c0_g3** | **DN62524_c2_g1** | **DN62524_c2_g2** |
| **Wisconsin (Hancock) vs Oregon** | 0.04 | <0.01 | 0.25 | 0.55 | 0.97 | 0.90 |
| **Wisconsin (Hancock) vs Wisconsin (Dane)** | 0.40 | <0.01 | 0.33 | 0.11 | 0.97 | 0.61 |
| **Wisconsin (Hancock) vs Maine (Arostook-2)** | 0.39 | <0.01 | 0.62 | 0.75 | 1.00 | 1.00 |
| **Wisconsin (Hancock) vs Maine (Arostook-1)** | 0.11 | <0.01 | 0.55 | 0.98 | 0.54 | 0.86 |
| **Maine (Arostook-1) vs Oregon** | 0.99 | 1.00 | <0.01 | 0.83 | 0.86 | 1.00 |
| **Maine (Arostook-1) vs Wisconsin (Dane)** | 0.93 | 0.98 | 0.01 | 0.25 | 0.18 | 0.12 |
| **Maine (Arostook-1) vs Maine (Arostook-2)** | 0.96 | 0.92 | 0.04 | 0.95 | 0.77 | 0.73 |
| **Maine (Arostook-2) vs Oregon** | 0.79 | 0.94 | 0.97 | 1.00 | 1.00 | 0.78 |
| **Maine (Arostook-2) vs Wisconsin (Dane)** | 1.00 | 0.64 | 0.99 | 0.70 | 0.84 | 0.77 |
| **Wisconsin (Dane) vs Oregon** | 0.70 | 0.96 | 1.00 | 0.83 | 0.69 | 0.14 |
|  |  |  |  |  |  |  |
|  | **DN62524_c2_g4** | **DN63738_c2_g1** |  |  |  |  |
| **Wisconsin (Hancock) vs Oregon** | 1.00 | <0.01 |  |  |  |  |
| **Wisconsin (Hancock) vs Wisconsin (Dane)** | 0.46 | <0.01 |  |  |  |  |
| **Wisconsin (Hancock) vs Maine (Arostook-2)** | 0.95 | <0.01 |  |  |  |  |
| **Wisconsin (Hancock) vs Maine (Arostook-1)** | 0.99 | <0.01 |  |  |  |  |
| **Maine (Arostook-1) vs Oregon** | 0.99 | 0.99 |  |  |  |  |
| **Maine (Arostook-1) vs Wisconsin (Dane)** | 0.19 | 0.98 |  |  |  |  |
| **Maine (Arostook-1) vs Maine (Arostook-2)** | 0.75 | 0.94 |  |  |  |  |
| **Maine (Arostook-2) vs Oregon** | 0.94 | 0.99 |  |  |  |  |
| **Maine (Arostook-2) vs Wisconsin (Dane)** | 0.87 | 1.00 |  |  |  |  |
| **Wisconsin (Dane) vs Oregon** | 0.40 | 0.89 |  |  |  |  |
