# Supplementary material for: Targeted RNA sequencing reveals differential patterns of transcript expression in geographically discrete, insecticide resistant populations of Leptinotarsa decemlineata
Source: Pest Manag Sci. 2021 May 3;77(7):3436–44. doi: 10.1002/ps.6393 (PMC8252485; doi:10.1002/ps.6393)
Supplement: Supplementary file 4 — Table S4. Mean transcript counts [file PS-77-3436-s002.docx]

**Supplemental Table S4.** Mean transcript counts, standard error, and Log2 fold change for each transcript.

|  | **Maine (Aroostook-1)** | | | **Maine (Aroostook-2)** | | | **Wisconsin (Hancock)** | | | **Wisconsin (Dane)** | | | **Oregon (Umatilla)** | | |
| --- | --- | --- | --- | --- | --- | --- | --- | --- | --- | --- | --- | --- | --- | --- | --- |
| **Transcript ID** | **Mean** | **STE** | **Log2** | **Mean** | **STE** | **Log2** | **Mean** | **STE** | **Log2** | **Mean** | **STE** | **Log2** | **Mean** | **STE** | **Log2** |
| DN52191_c2_g3 | 17.96 | 11.06 | 4.17 | 0.49 | 0.21 | -1.02 | 22.81 | 10.55 | 4.51 | 30.80 | 14.81 | 4.95 | 14.89 | 9.43 | 3.90 |
| DN45930_c0_g1 | 14.74 | 8.54 | 3.88 | 0.86 | 0.32 | -0.22 | 31.83 | 6.65 | 4.99 | 22.40 | 9.86 | 4.49 | 12.70 | 8.03 | 3.67 |
| DN51839_c1_g1 | 14.25 | 5.17 | 3.83 | 20.44 | 16.88 | 4.35 | 68.24 | 10.27 | 6.09 | 13.77 | 2.80 | 3.78 | 6.12 | 1.66 | 2.61 |
| DN45995_c0_g1 | 2.45 | 0.78 | 1.29 | 1.42 | 0.58 | 0.50 | 11.81 | 1.55 | 3.56 | 3.82 | 1.43 | 1.93 | 1.71 | 0.57 | 0.77 |
| DN46083_c0_g3 | 15.28 | 9.20 | 3.93 | 0.88 | 0.33 | -0.18 | 39.68 | 21.56 | 5.31 | 12.27 | 7.12 | 3.62 | 29.28 | 18.10 | 4.87 |
| DN47979_c8_g1 | 7.86 | 2.92 | 2.97 | 6.01 | 3.74 | 2.59 | 47.41 | 8.35 | 5.57 | 5.95 | 1.61 | 2.57 | 7.46 | 4.13 | 2.90 |
| DN43906_c0_g1 | 22.10 | 5.99 | 4.47 | 11.03 | 7.97 | 3.46 | 75.26 | 11.16 | 6.23 | 12.21 | 3.08 | 3.61 | 5.23 | 2.20 | 2.39 |
| DN61141_c1_g1 | 59.50 | 20.69 | 5.89 | 13.64 | 5.68 | 3.77 | 36.18 | 5.23 | 5.18 | 6.97 | 2.69 | 2.80 | 4.29 | 1.06 | 2.10 |
| comp106072_c0 | 7.45 | 0.95 | 2.90 | 8.95 | 0.58 | 3.16 | 13.70 | 1.79 | 3.78 | 9.21 | 1.04 | 3.20 | 9.52 | 3.10 | 3.25 |
| comp111691_c1 | 9.69 | 3.87 | 3.28 | 5.09 | 2.66 | 2.35 | 62.43 | 15.52 | 5.96 | 8.87 | 1.36 | 3.15 | 3.55 | 1.20 | 1.83 |
| comp103658_c0 | 3.33 | 0.38 | 1.73 | 3.91 | 0.24 | 1.97 | 5.88 | 0.54 | 2.56 | 4.04 | 0.43 | 2.02 | 3.59 | 1.04 | 1.85 |
| DN33393_c0_g1 | 8.37 | 4.13 | 3.07 | 2.38 | 1.73 | 1.25 | 31.72 | 6.52 | 4.99 | 17.57 | 7.98 | 4.14 | 10.44 | 6.52 | 3.38 |
| DN44684_c0_g1 | 7.95 | 4.48 | 2.99 | 0.63 | 0.30 | -0.67 | 9.88 | 3.56 | 3.30 | 10.78 | 4.82 | 3.43 | 6.18 | 3.72 | 2.63 |
| DN61595_c0_g3 | 15.60 | 5.32 | 3.96 | 11.31 | 2.22 | 3.50 | 18.86 | 2.37 | 4.24 | 3.56 | 0.79 | 1.83 | 9.60 | 6.28 | 3.26 |
| comp114026_c0 | 1.11 | 0.10 | 0.15 | 0.76 | 0.15 | -0.39 | 1.78 | 0.14 | 0.83 | 1.41 | 0.22 | 0.49 | 0.96 | 0.22 | -0.07 |
| DN52951_c2_g1 | 3.53 | 0.70 | 1.82 | 3.73 | 0.40 | 1.90 | 9.48 | 2.09 | 3.24 | 4.72 | 0.67 | 2.24 | 4.07 | 1.43 | 2.03 |
| comp117371_c0 | 4.74 | 0.62 | 2.25 | 2.97 | 0.73 | 1.57 | 5.72 | 0.37 | 2.52 | 5.28 | 0.48 | 2.40 | 4.01 | 1.42 | 2.00 |
| comp117821_c0 | 10.67 | 1.64 | 3.42 | 5.01 | 1.22 | 2.32 | 13.24 | 2.08 | 3.73 | 12.94 | 1.85 | 3.69 | 7.71 | 2.50 | 2.95 |
| comp118021_c0 | 3.29 | 0.50 | 1.72 | 1.75 | 0.55 | 0.80 | 2.04 | 0.49 | 1.03 | 3.42 | 0.83 | 1.77 | 4.43 | 1.79 | 2.15 |
| DN63738_c2_g1 | 9.44 | 2.24 | 3.24 | 6.70 | 1.63 | 2.74 | 33.94 | 3.81 | 5.08 | 6.34 | 0.90 | 2.66 | 8.11 | 3.11 | 3.02 |
| DN48293_c3_g1 | 3.74 | 0.73 | 1.90 | 2.86 | 0.44 | 1.51 | 5.86 | 0.69 | 2.55 | 4.49 | 0.80 | 2.17 | 3.66 | 1.13 | 1.87 |
| DN48864_c1_g1 | 10.55 | 3.34 | 3.40 | 3.93 | 2.62 | 1.97 | 38.24 | 5.70 | 5.26 | 17.98 | 7.36 | 4.17 | 6.38 | 3.23 | 2.67 |
| DN41892_c0_g1 | 4.40 | 0.73 | 2.14 | 8.71 | 1.01 | 3.12 | 6.99 | 0.90 | 2.81 | 8.29 | 2.84 | 3.05 | 4.27 | 0.90 | 2.09 |
| DN62524_c2_g2 | 2.24 | 0.36 | 1.16 | 3.68 | 0.74 | 1.88 | 3.37 | 0.36 | 1.75 | 5.04 | 1.41 | 2.33 | 2.35 | 0.51 | 1.23 |
| DN62524_c2_g4 | 4.67 | 0.96 | 2.22 | 10.99 | 3.13 | 3.46 | 7.13 | 0.69 | 2.83 | 15.98 | 6.73 | 4.00 | 7.03 | 2.06 | 2.81 |
| DN45929_c0_g1 | 15.54 | 8.67 | 3.96 | 0.97 | 0.31 | -0.05 | 25.51 | 7.06 | 4.67 | 23.76 | 10.64 | 4.57 | 12.03 | 7.23 | 3.59 |
| DN62524_c2_g1 | 4.45 | 0.82 | 2.15 | 6.83 | 0.56 | 2.77 | 7.60 | 0.84 | 2.93 | 8.91 | 2.43 | 3.16 | 6.37 | 1.20 | 2.67 |
| DN54580_c0_g1 | 10.73 | 6.03 | 3.42 | 1.69 | 0.81 | 0.75 | 15.76 | 5.57 | 3.98 | 21.25 | 9.57 | 4.41 | 6.60 | 4.00 | 2.72 |
| DN56141_c0_g1 | 2.73 | 0.65 | 1.45 | 3.90 | 1.68 | 1.96 | 7.24 | 2.21 | 2.86 | 4.08 | 0.88 | 2.03 | 1.92 | 0.42 | 0.94 |
| DN48501_c1_g1 | 25.50 | 3.30 | 4.67 | 12.56 | 3.93 | 3.65 | 18.60 | 1.99 | 4.22 | 12.49 | 1.98 | 3.64 | 3.55 | 1.31 | 1.83 |
| comp114343_c0 | 1.92 | 0.61 | 0.94 | 1.41 | 0.23 | 0.49 | 3.53 | 0.79 | 1.82 | 3.74 | 0.55 | 1.90 | 3.78 | 0.71 | 1.92 |
| DN53725_c1_g1 | 13.61 | 4.57 | 3.77 | 8.07 | 5.14 | 3.01 | 113.19 | 8.89 | 6.82 | 29.16 | 12.72 | 4.87 | 5.48 | 1.55 | 2.45 |
| DN48928_c1_g1 | 1.67 | 0.38 | 0.74 | 0.65 | 0.28 | -0.63 | 8.19 | 1.47 | 3.03 | 3.82 | 1.97 | 1.93 | 0.65 | 0.20 | -0.63 |
| DN23859_c0_g1 | 7.59 | 3.79 | 2.92 | 1.87 | 1.13 | 0.90 | 20.88 | 4.31 | 4.38 | 14.16 | 9.86 | 3.82 | 6.13 | 3.41 | 2.62 |
| DN42933_c0_g1 | 5.28 | 3.41 | 2.40 | 0.39 | 0.14 | -1.34 | 10.93 | 3.91 | 3.45 | 15.10 | 6.64 | 3.92 | 4.99 | 2.66 | 2.32 |
| DN45742_c0_g1 | 27.03 | 22.56 | 4.76 | 2.59 | 0.48 | 1.37 | 16.07 | 7.68 | 4.01 | 1.71 | 0.34 | 0.78 | 0.28 | 0.07 | -1.84 |
| DN59030_c2_g1 | 11.93 | 4.13 | 3.58 | 5.59 | 4.05 | 2.48 | 57.45 | 4.66 | 5.84 | 16.24 | 6.13 | 4.02 | 11.37 | 6.45 | 3.51 |
| DN44960_c0_g1 | 2.60 | 1.04 | 1.38 | 2.76 | 1.98 | 1.46 | 13.80 | 3.34 | 3.79 | 1.98 | 0.47 | 0.99 | 0.71 | 0.25 | -0.48 |
