# Supplementary material for: Targeted RNA sequencing reveals differential patterns of transcript expression in geographically discrete, insecticide resistant populations of Leptinotarsa decemlineata
Source: Pest Manag Sci. 2021 May 3;77(7):3436–44. doi: 10.1002/ps.6393 (PMC8252485; doi:10.1002/ps.6393)
Supplement: Supplementary file 5 — Table S5. Quantitative PCR primers and primer efficiencies. [file PS-77-3436-s006.docx]

**Supplemental Table S5:** Quantitative PCR primers and primer efficiencies.

| **Forward Primer (5’-3’)** | **Reverse Primer (5’-3’)** | **Primer Efficiency** | **Targeted Gene** |
| --- | --- | --- | --- |
| AAAGAAACGAGCATTGCCCTTCCG | TTGTCGCTGACACTGTAGGGTTGA | 1.93 | Ribosomal protein 4 |
| TGCTGAAAGACCTGGAAGTG | CTCATCATGGGAAGAAGACTGG | 2.01 | Cytochrome P450 6k1 |
| TCCGCAACCTCCATCTTTATAC | AACCTGACCCAAAGATAGTACAG | 1.88 | UDP-glucuronosyltransferase 2B10-like |
