# Supplementary material for: Targeted RNA sequencing reveals differential patterns of transcript expression in geographically discrete, insecticide resistant populations of Leptinotarsa decemlineata
Source: Pest Manag Sci. 2021 May 3;77(7):3436–44. doi: 10.1002/ps.6393 (PMC8252485; doi:10.1002/ps.6393)

**Supplemental Figure S1:** Principle component analysis of examined transcript expression by location.


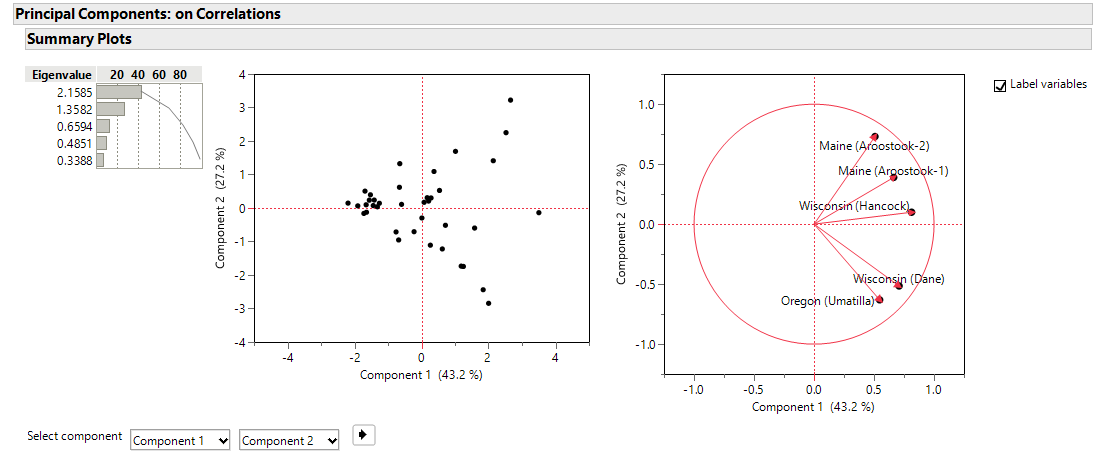

Supplement: Supplementary file 6 — Figure S1. Principle component analysis of examined transcript expression by location [file PS-77-3436-s004.docx]
